# The Mismatch Between Professionally Produced Vaccine Content and Audience Demand on Chinese Short-Form Video Platforms: A Cross-Platform Content Analysis

**DOI:** 10.3390/vaccines14060491

**Published:** 2026-05-30

**Authors:** Yuqi Fu, Yuan Dang, Yuming Liu, Yangmu Huang

**Affiliations:** 1 School of Public Health, Peking University, Beijing 100191, China; 2210306144@stu.pku.edu.cn; 2 Department of Global Health, School of Public Health, Peking University, Beijing 100191, China; 2211210114@bjmu.edu.cn (Y.D.); 2411110222@stu.pku.edu.cn (Y.L.)

**Keywords:** vaccine communication, vaccine hesitancy, short-form video platforms, social media engagement, content analysis, audience demand, China

## Abstract

**Background**: Short-form video platforms have become important channels for vaccine science communication, yet whether professionally produced vaccine content aligns with audience demand remains underexplored. **Methods**: We conducted a cross-sectional quantitative content analysis of 3752 publicly available vaccine-related videos retrieved from three major Chinese short-form video platforms between 21 November and 13 December 2024. A coding framework based on the Health Belief Model (HBM) and the World Health Organization (WHO) Behavioral and Social Drivers (BeSD) framework was used to identify key content themes. Multivariate Bayesian negative binomial regression and demand–avoidance analysis were used to examine engagement patterns and supply–demand alignment across account types. **Results**: Individual users produced the majority of videos (53.17%), whereas medical professionals received the highest level of engagement. Engagement was positively associated with themes related to disease severity (β ≈ 0.19–0.25) and side effects and management (β ≈ 0.31–0.67), but negatively associated with vaccine effectiveness (β ≈ −0.28 to −0.14) and vaccination precautions (β ≈ −0.28 to −0.27). Professional sources showed broader thematic coverage but also the greatest supply–demand mismatch, with mismatch indices of 0.377 for medical institution official media and 0.304 for medical professionals, primarily driven by overrepresentation of themes associated with audience avoidance. **Conclusions**: Significant structural mismatch exists between professionally produced vaccine content and audience engagement-based demand on short-form video platforms. Optimizing vaccine communication may require prioritizing audience-concerned risk-related information and dynamically adjusting content strategies based on engagement feedback to enhance the effectiveness of vaccine education.

## 1. Introduction

Vaccination is widely recognized as one of the most important public health interventions for preventing infectious diseases [1,2]. However, persistent gaps in vaccine uptake indicate that availability alone is insufficient to ensure widespread immunization [3]. In China, several voluntary vaccines continue to exhibit suboptimal uptake across different populations. Many studies have reported vaccine hesitancy and refusal rates ranging from 10% to 30%, while vaccination coverage for multiple vaccines remains below 30% [4,5,6,7,8]. Even when vaccines are safe and available, hesitancy shaped by misinformation, risk perceptions, trust deficits, and limited health literacy can substantially influence immunization decisions [1,2,9,10]. This underscores the growing importance of how vaccine information is communicated, rather than merely its availability.

Social media has become a major platform for disseminating health information, offering new opportunities to influence vaccination attitudes and behaviors [11,12]. Evidence from multiple studies shows that targeted interventions delivered through social media can improve vaccine knowledge, increase vaccination intentions, and counter misinformation [12,13,14,15,16]. Among various social media formats, short-form video platforms are particularly influential due to their rapid dissemination, audiovisual appeal, and algorithm distribution. In China, highly developed and large-scale short-form video platforms such as Douyin (TikTok China), Kuaishou (Kwai), and Xiaohongshu (Rednote) have attracted large user bases, with approximately 850 million, 410 million, and 350 million daily active users, respectively [17]. These platforms have become important channels for public health communication and provide a valuable empirical setting for examining vaccine communication in algorithm-driven short-form video environments [18,19].

These platforms host a diverse ecosystem of content producers with varying levels of expertise, institutional authority, and communication goals. Differences in producers’ backgrounds and communication goals shape narrative strategies, reflected in variations in style, tone, informational accuracy, and structure. Prior research indicates that misinformation and negatively framed vaccine narratives can undermine public perceptions and attitudes toward vaccination [20,21,22,23]. Healthcare professionals and official institutional accounts are generally regarded as authoritative sources of vaccine-related information, and their content and language style are often perceived as more credible and professionally rigorous [24,25,26,27,28], potentially eliciting positive responses and strengthening vaccination intentions [25,29,30,31,32,33,34]. However, professional authority alone does not guarantee audience engagement or resonance in short-form video environments. More comprehensive or technically detailed content does not necessarily generate higher user engagement [35]. The structure and framing of vaccine information shape how audiences interpret risk and make health decisions [36], yet even accurate content may fail to attract attention in algorithm-driven feeds.

Despite these insights, three important gaps remain in the existing literature. First, although prior studies acknowledge the diversity of content producers, systematic comparative analyses of how different producer types vary in their thematic supply of vaccine information remain limited. Second, while some studies examine engagement-related factors, the relationship between specific vaccine-related themes and audience engagement remains insufficiently examined in a systematic and quantitative manner. Third, empirical evidence directly quantifying whether the thematic supply of professionally produced vaccine content aligns with audience demand remains scarce, particularly regarding supply–demand mismatch in short-form video environments.

To systematically characterize the structure of vaccine-related short-form videos, a theoretically grounded framework is needed to identify key informational cues. The Health Belief Model (HBM) conceptualizes vaccination decisions in terms of perceived susceptibility and severity, perceived benefits and risks, and perceived barriers to action [37]. Complementing this individual-level perspective, the Behavioral and Social Drivers (BeSD) framework incorporates broader social and contextual determinants, including trust, social norms, motivation, and access [38]. Together, these frameworks provide a structured basis for identifying vaccination-relevant information cues. In this study, they are used to organize and classify thematic content in short-form videos, rather than to directly infer individual vaccination behavior [32,39,40,41].

Short-form video platforms provide multiple engagement metrics that quantify how audiences interact with content. Users interact with content through observable actions such as likes, shares, favorites, and comments, and these engagement metrics are commonly used to evaluate the effectiveness of online health communication [39,42]. Although these indicators do not directly measure actual vaccination intentions, normative health information needs, or behavior change, they capture observable patterns of audience responsiveness and content preference [43,44]. In this study, “audience demand” refers specifically to engagement-based audience responsiveness on short-form video platforms, rather than normative health information needs. While engagement metrics may also be shaped by algorithm-driven recommendation mechanisms and broader attention dynamics, they remain useful behavioral indicators for examining the alignment between content supply and audience engagement patterns in algorithm media environments [45].

This cross-platform content analysis examines how vaccine-related content themes are distributed across account types and how these themes relate to audience engagement, using engagement metrics and thematic coverage as analytic indicators. By identifying supply–demand mismatches, the study aims to generate empirical evidence for more targeted and effective vaccine communication strategies and to support efforts to improve vaccine information access and understanding.

## 2. Materials and Methods

### 2.1. Data Source and Sampling

Vaccine-related videos were retrieved from Xiaohongshu, Douyin, and Kuaishou between 21 November and 13 December 2024, using a keyword-based search strategy. No restriction was imposed on the original publication date of videos. Search terms included general vaccine terms (e.g., “疫苗 [vaccine]”, “疫苗接种 [vaccination]”), descriptive terms (e.g., “疫苗副作用 [vaccine side effects]”, “疫苗安全性 [vaccine safety]”), and specific vaccines (e.g., “HPV疫苗 [HPV vaccine]”, “九价疫苗 [9-valent HPV vaccine]”). The full list of search terms is provided in Supplementary Table S1. Raw video metadata were retrieved using automated scripts based on Python (version 3.12, 64-bit; Python Software Foundation, Wilmington, DE, USA) through platform Application Programming Interface (API) access.

Extracted variables were organized using a structured data extraction spreadsheet developed in Microsoft Excel (Office LTSC 2024; Microsoft Corporation, Redmond, WA, USA). Variables were determined through discussions within the research team prior to formal extraction and included information directly obtainable during the extraction process, such as video title, text content generated via speech-to-text transcription, engagement metrics (likes, favorites, shares, comments), and account-level metrics such as followers and the total likes received by the account.

Subsequent preprocessing was conducted in Microsoft Excel, including deduplication, manual verification, eligibility screening, and exclusion of records with incomplete analytical variables to improve data consistency and accuracy prior to analysis. Videos were excluded if they met any of the following criteria: duplicate content, non-Chinese content, non-human vaccine-related content, content not primarily related to vaccination, advertisements, or COVID-19–related content. The final analytic dataset included 3752 videos.

Each engagement variable was winsorized separately at the 99th percentile to reduce the influence of extreme outliers while preserving the overall distributional pattern. Results remained substantively unchanged in sensitivity analyses without winsorization.

### 2.2. Measures

#### 2.2.1. Independent Variable

Videos were classified into five account types, including medical professionals, medical institution official media, non-medical institution official media, private-sector accounts, and individual users. Account types were classified based on profile verification, self-descriptions, and observable institutional affiliations. Medical professionals were defined as individuals with a background in medical knowledge and clear involvement in medical-related professions. Medical institution official media were defined as accounts representing hospitals, clinics, Centers for Disease Control and Prevention (CDC) and other public health agencies, or other healthcare organizations. Non-medical institution official media were defined as verified accounts of government agencies (non-health), universities, and authoritative public news outlets. Private-sector accounts were defined as accounts with explicit commercial intent, such as product marketing, service promotion, or monetized vaccine-related content.

#### 2.2.2. Dependent Variable

Engagement metrics were used as the dependent variables, including the number of likes, shares, favorites, and comments. These metrics capture attention and interaction rather than behavioral outcomes and were therefore used as indicators of engagement-based content demand. Because view counts were not consistently available and are not directly comparable across platforms due to differences in platform-specific counting mechanisms and algorithmic exposure, the present study focused on four observable engagement indicators that more directly reflect user interaction. These variables were analyzed as raw count data and were winsorized at the 99th percentile prior to analysis, as described in the Data Preprocessing Section.

#### 2.2.3. Covariates

We included video format (personal experience, lifestyle sharing, health education, and hot-topic news) and vaccine type (National Immunization Program vaccines [NIP], non-NIP vaccines, both vaccine types, or other/unspecified) as covariates to adjust for presentation style and vaccine attributes in engagement outcomes. To capture account-level influence, follower count and the total likes received by the account were log-transformed, standardized, and combined into a social influence index using a latent variable approach.

### 2.3. Content Coding Framework

We adopted a hybrid approach that combined theory-informed and data-driven procedures to develop the coding framework. Initial codes were guided by the HBM and the BeSD framework. We refined the scheme iteratively based on observed discourse patterns, resulting in a final set of ten codes (details are listed in Supplementary Table S2). The codes covered perceived susceptibility and severity; vaccine effectiveness and mechanism of action; safety concerns (including adverse effects and management); and practical issues such as accessibility and affordability, and vaccination procedures. Meanwhile, negative narratives and health myths were coded as indicators to capture negative vaccine discourse. Coding was conducted at the sentence level. Each theme was treated as an independent signal, and the frequency of each was operationalized as a quantitative variable. Content frequency was winsorized at the 99th percentile to mitigate the influence of extreme values, enabling subsequent statistical analysis of vaccination-related discourse on short-form video platforms.

To enhance coding consistency, two researchers independently reviewed a random 10% subsample during the initial coding stage using the predefined codebook. Agreement exceeded 95% across reviewed coding items, indicating high coding consistency. Discrepancies were discussed and resolved through consensus, and the coding scheme was refined iteratively before application to the full dataset. Formal intercoder reliability statistics were not calculated.

### 2.4. Statistical Analysis

Descriptive statistics were used to summarize the distributions of publisher account types, content themes, and engagement outcomes. Differences in engagement across account categories were assessed using Kruskal–Wallis tests, with significance set at an α of 0.05.

Multivariate Bayesian negative binomial regression models were used because the engagement outcomes were overdispersed count variables, and the Bayesian framework with Markov chain Monte Carlo (MCMC) estimation enabled stable parameter estimation and uncertainty quantification, particularly for subgroup analyses. Prior to Bayesian modeling, separate univariate negative binomial regression analyses were conducted for each engagement outcome, and covariates showing significant associations were subsequently included in the corresponding multivariable models. Candidate covariates included account types, video types, vaccine types, social influence index, and frequencies of content themes, and final adjusted models were simplified to improve model parsimony while retaining substantively relevant covariates. Analyses were first conducted on the full dataset and then stratified by account type to examine heterogeneity in theme–engagement associations across producer groups and to support subsequent mismatch analyses. Although subgroup analyses were performed for all account types, the present study primarily reports results for medical professionals and medical institution official media due to their central relevance to professional vaccine communication. Uncertainty was quantified using 95% credible intervals derived from posterior distributions rather than dichotomous null hypothesis significance testing. Therefore, formal multiple-comparison correction procedures were not applied. Detailed model specifications, priors, and MCMC diagnostics are provided in Supplementary Method S1.

Based on the regression results, a demand–avoidance analysis was conducted to describe the alignment between content supply and audience engagement across publishing account types. Positive regression coefficients were interpreted as signals of higher audience engagement and potential audience interest, whereas negative coefficients indicated avoidance. To account for variation across engagement metrics, classification was based on both the consistency and magnitude of effects. Specifically, themes showing positive or negative associations across at least two engagement outcomes and with an average effect size ≥ 0.2 were classified as high demand or high avoidance, respectively. Less consistent or weaker patterns were categorized as moderate or low levels. Furthermore, weighted indices were calculated to quantify account-level demand gap or avoidance redundancy, and the overall mismatch index across account types. These indices were constructed as relative comparative measures within the study sample rather than standardized scales with predefined thresholds. Detailed definitions and calculation procedures are provided in Supplementary Method S2.

All statistical analyses and visualizations were conducted in R (version 4.4.3; R Foundation for Statistical Computing, Vienna, Austria).

## 3. Results

The final dataset comprised 3752 vaccine-related videos. We first describe the distribution of vaccine content producers, followed by comparisons of thematic supply across account types, and finally assess how thematic structures are associated with audience engagement and supply–demand mismatch.

### 3.1. Who Provides Vaccine Information and Who Gains Engagement

Individual users constituted the largest proportion of content providers, accounting for 53.17% (*n* = 1995) of all videos. Medical professionals (21.62%, *n* = 811) and non-medical institution official media (19.77%, *n* = 742) formed a second tier of contributors. In contrast, medical institution official media (3.65%, *n* = 137) and private-sector accounts (1.79%, *n* = 67) only accounted for a small share of videos.

**Figure 1:**
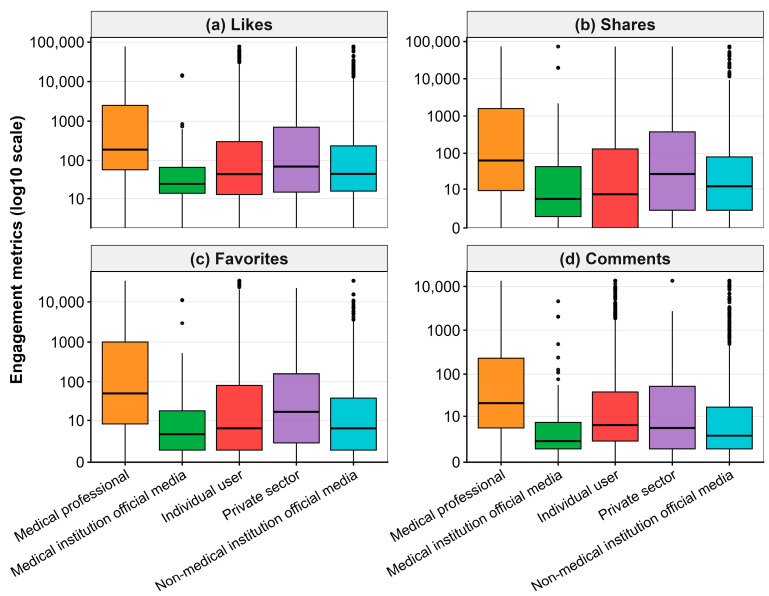
Box plots of the distribution of user engagement metrics. Four engagement metrics are presented separately by facets: (**a**) Likes, (**b**) Shares, (**c**) Favorites, and (**d**) Comments. The *y*-axis is log10-transformed with a + 1 offset (log_10_(x + 1)) to accommodate zero values and the highly right-skewed distribution of engagement data. For each box plot, the horizontal line within the box indicates the median value, the box boundaries represent the 25th and 75th percentiles (IQR), whiskers extend to 1.5 × IQR, and dots denote outlier values beyond this range.

Audience engagement differed significantly by the source of vaccine-related information across all engagement metrics. As shown in Table 1 and Figure 1, median engagement differed significantly across producer categories for all four metrics (Kruskal–Wallis tests: all *p* < 0.001). Medical professional accounts achieved the highest median engagement across all four metrics: 190 likes (IQR, 68–2498), 63 shares (IQR, 9–1582), 51 favorites (IQR, 8–1002), and 21 comments (IQR, 5–229.5). Despite producing fewer videos, medical professionals consistently generated higher engagement, indicating that content source may play a more important role than sheer content volume. By contrast, medical institution official accounts showed the lowest medians: 25 likes (IQR, 14–67), 5 shares (IQR, 1–43), 4 favorites (IQR, 1–18), and 2 comments (IQR, 1–7), approximately one-fourth to one-eighth of those achieved by medical professional accounts, suggesting that higher institutional authority did not necessarily correspond to higher audience engagement on these platforms. Among the remaining account types, including individual users, private-sector accounts, and non-medical institution official media, engagement patterns varied by metric, but overall medians were between the two extremes. Engagement distributions were highly right-skewed, as reflected in Figure 1.

### 3.2. What Content Structures Do Different Producers Supply

**Figure 2:**
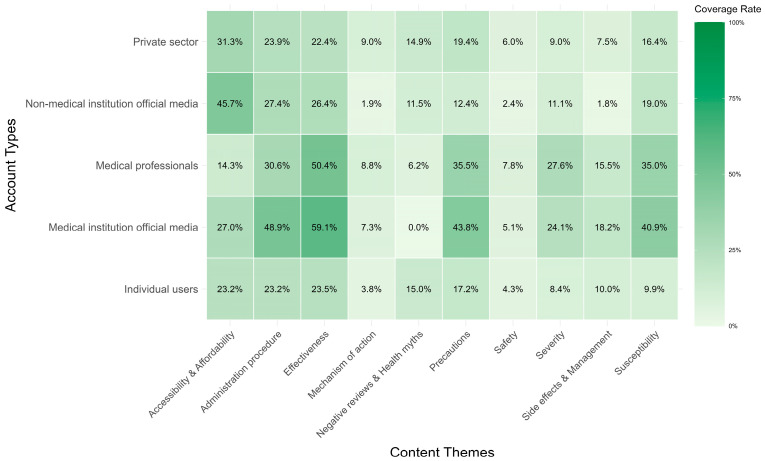
The distribution of coverage of vaccine-related content themes across account types. The heatmap shows the proportion of videos covering each content theme within different account types. Values represent the percentage of videos addressing each theme, and darker green colors indicate higher coverage rates.

Theme coverage differed across producer types, as shown in the theme coverage rates presented in Figure 2 (where coverage is defined as the share of videos containing at least one sentence coded for a given theme). Overall coverage levels were relatively modest, with only a limited number of themes exceeding 40% coverage within specific producer groups. In general, producers most frequently covered susceptibility, effectiveness, accessibility and affordability, and vaccination procedures and precautions, whereas mechanism of action and safety themes were comparatively less frequently addressed.

Medical institution official media and medical professionals showed broader thematic coverage than other account types, suggesting a wider informational scope. Across all account types, medical institution official media showed the highest coverage of themes related to disease susceptibility (40.9%), vaccine effectiveness (59.1%), vaccination procedures (48.9%), vaccination precautions (43.8%), and side effects and management (18.2%). By contrast, medical professionals showed the highest coverage of disease severity (27.6%) and vaccine safety (7.8%).

Private-sector accounts, individual users, and non-medical institution official media all emphasized effectiveness, accessibility and affordability, and vaccination procedures. Differences in thematic emphasis were observed, with non-medical institution official media and private-sector accounts placing greater emphasis on accessibility and affordability (45.7% and 31.3%, respectively).

Notably, broader thematic coverage did not necessarily correspond to stronger audience engagement, underscoring a potential mismatch between content supply and engagement-based audience demand, which is examined in the following section.

### 3.3. How Content Structures Translate into Engagement Outcomes

**Figure 3:**
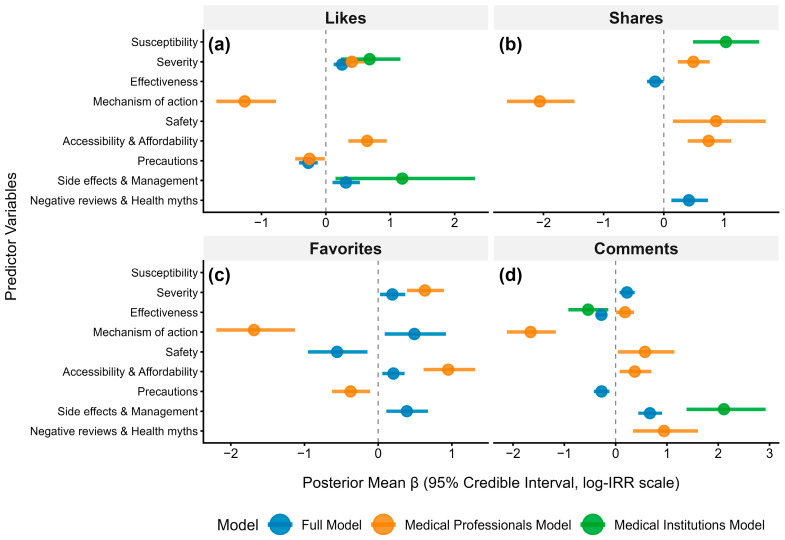
The effects of vaccine-related content themes on user engagement across four engagement metrics. (**a**) Likes, (**b**) Shares, (**c**) Favorites, and (**d**) Comments. Forest plots display posterior mean coefficients (log scale) and 95% credible intervals from multivariate Bayesian negative binomial regression models predicting four engagement metrics (likes, shares, favorites, and comments). Colors represent results for the full sample (blue), medical professionals (orange), and medical institution official media (green), and the dashed vertical line indicates the null effect (β = 0). Positive and negative coefficients indicate relatively higher and lower expected engagement, respectively. Full results are provided in Supplementary Tables S3–S5 and Supplementary Figure S1.

Full-sample regression models revealed differentiated engagement responses across content themes (Figure 3). Complete regression results for the full sample are provided in Supplementary Table S3. Specifically, severity (β ≈ 0.19–0.25) and side effects and management (β ≈ 0.31–0.67) were positively associated with engagement, indicating stronger audience responses to risk-related information. In contrast, effectiveness (β ≈ −0.28 to −0.14) and precautions (β ≈ −0.28 to −0.27) showed consistently negative associations across multiple outcomes. Coefficients are reported on the log scale and therefore reflect the direction and relative magnitude of associations rather than absolute changes in engagement, with larger absolute coefficient values indicating stronger relative associations. By comparison, themes such as mechanism of action and safety showed weaker or less consistent associations across engagement metrics, suggesting more limited or context-dependent audience responses.

Associations between content themes and engagement varied substantially across medical professional and medical institution official media accounts (Figure 3). Although subgroup analyses were conducted for all account types, the results presented here focus on medical professionals and medical institution official media, in line with the study’s emphasis on professional content supply. In medical professional accounts, severity (β ≈ 0.40–0.63), safety (β ≈ 0.57–0.87), and accessibility and affordability (β ≈ 0.37–0.95) were positively associated with engagement. In contrast, mechanism of action content (β ≈ −2.06 to −1.26) and precautions (β ≈ −0.37 to −0.25) showed negative associations, suggesting that highly technical or procedural content may be less aligned with audience engagement preferences in this subgroup.

By comparison, associations between content themes and engagement metrics were more fragmented among videos published by medical institution official media, with only a subset of themes showing clear associations with specific engagement outcomes. Severity content remained positively associated with likes (β = 0.68). Side effects and management showed stronger positive associations with both likes and comments (β ≈ 1.19–2.11). By contrast, vaccine effectiveness-themed content was negatively associated with engagement in this subgroup (β = −0.54).

Detailed subgroup-specific estimates are provided in Supplementary Tables S4 and S5. Full model outputs, including covariates and posterior distributions across engagement metrics, are presented in Supplementary Figure S1.

### 3.4. Mismatch Between Professional Supply and Audience Engagement-Based Demand

A clear mismatch was observed between thematic content supply and engagement-based audience demand, particularly among professional sources (Table 2). Medical institution official media and medical professionals exhibited the largest mismatches within the sample, with mismatch indices of 0.377 and 0.304, respectively. These values were higher than those for individual users (0.120), private-sector accounts (0.094), and non-medical institution official media (0.068). As the mismatch index is a relative measure constructed within the study sample, higher values indicate greater divergence between thematic content supply and engagement-based audience demand across account types. Notably, the higher mismatch observed among professional sources was driven primarily by overrepresentation of themes associated with audience avoidance rather than insufficient coverage of demand-related themes.

Further decomposition of mismatch sources indicated that, despite relatively high coverage of demand themes, professional producers also overrepresented themes associated with audience avoidance, which largely drove their higher mismatch indices. In both professional groups, the weighted demand gap rate was close to zero, whereas the corresponding avoidance redundancy index was higher than that of other account types. In contrast, individual users and private-sector accounts showed a moderate demand gap but limited avoidance redundancy, resulting in comparatively lower mismatch.

At the theme level, mismatch patterns differed between medical institution official media and medical professional accounts. For medical institution official media, under-supplied themes clustered around severity, and around side effects and management, whereas effectiveness emerged as the dominant avoidance theme. Among medical professionals, under-supplied themes mainly involved severity, accessibility and affordability, and safety, whereas precautions showed pronounced redundancy. Overall, mismatch among professional sources was primarily driven by overrepresentation of avoidance-related themes.

## 4. Discussion

This study shows that professionally produced vaccine communication on Chinese short-form video platforms is characterized by a clear structural mismatch between content supply and audience engagement patterns. Although professional sources provide broader thematic coverage, they do not necessarily prioritize the themes most strongly associated with user engagement. This finding extends existing vaccine communication research by demonstrating that informational breadth and professional authority do not automatically translate into audience resonance in algorithm-driven environments.

In this study, medical professionals and medical institution official media accounted for a relatively small share of total videos compared to individual users. This distribution is broadly consistent with prior research across multiple social media platforms and geographic contexts, including studies on Weibo in China and TikTok in Italy as well as international samples [26,46,47]. Rather than simply reflecting differences in participation, this distribution highlights a structural imbalance in short-form video production, where professional sources contribute less frequently but play a central role in shaping health information.

Although prior research often groups medical professionals and medical institution official media together, our findings suggest they should be considered separately in short-video vaccine communication research. Medical professionals achieved the highest engagement, whereas medical institution official media received the lowest. Similar patterns have been reported in prior studies [23,32,47,48,49,50]. Even within professional sources, organizational form, communication goals, and language style may lead to markedly different engagement outcomes [29,51].

At the thematic level, the two professional producer types displayed broadly similar supply structures. Within short-form video environments, engagement was consistently concentrated on risk-oriented themes, especially severity, safety, and side effects and management. System-oriented themes (e.g., effectiveness, mechanism of action, and procedures) were associated with lower engagement levels. This risk-oriented pattern aligns with previous work showing that while medical professionals tend to emphasize procedures and benefits [52], individual creators more often foregrounded safety concerns [53]. Furthermore, these insights are consistent with prior research indicating that concerns about safety and side effects are major drivers of vaccine hesitancy across settings [1,10,29,39,54,55,56], which may help explain why audiences engage more with safety and side effects and management than with effectiveness-focused content [29]. Public concerns about vaccine safety are often shaped by professional recommendations [1,9]. At the same time, misinformation about adverse events circulates widely online [57]. Professional explanations of side effects may therefore play a critical role in reducing public anxiety and uncertainty [10,20,58], underscoring the importance of prioritizing high-demand themes in professionally produced vaccine content.

Compared with the relatively fragmented demand patterns observed for medical institution official media, audiences engaging with medical professional accounts showed clearer thematic preferences. Engagement increased substantially when risk-oriented themes were addressed. This suggests that qualities such as personal credibility, experiential narratives, and more relatable communication styles may allow individual medical professionals to present evidence-based information in ways that better activate emotional responses and risk perception in algorithm-driven environments [9,10,23,29,39]. These dynamics are consistent with prior reviews highlighting the role of communication strategies in shaping vaccine attitudes [29,32]. However, medical professional video producers may not fully leverage their engagement advantage if their content does not prioritize audiences’ primary concerns. This reflects a structural mismatch in how communication priorities are allocated.

In contrast, medical institution official media showed a broader pattern of supply–demand mismatch, characterized by low overall engagement and limited theme-specific associations. Both professional producer types tended to prioritize comprehensive coverage and system-oriented information. More fundamentally, their communication often follows an organizationally structured and standardized logic, shaped by clinical training, regulatory expectations, and traditional health education models that emphasize informational completeness and scientific accuracy while paying less attention to communication techniques [59,60,61]. However, this system-oriented communication logic may not align well with the attention competition dynamics of short-form video platforms. In algorithm-driven feeds, content visibility depends on rapid attention capture, emotional salience, and user interaction. As a result, informationally dense and system-oriented content may be less effective in attracting engagement compared to more focused, risk-oriented messaging. Such informationally dense communication may also contribute to information overload, reducing the audience’s willingness to engage and limiting dissemination [36,58,62]. In this context, informational completeness may be necessary for professional credibility but insufficient for audience engagement. Professional authority or identity alone is therefore insufficient to ensure alignment between content supply and engagement-based demand. Effective engagement depends on whether communication strategically prioritizes themes that resonate with audience concerns. Theme selection and emphasis, and their alignment with audience concerns, appear to be decisive factors [50]. These findings highlight heterogeneity within professional sources and inform how to optimize content strategies while maintaining professional credibility. The multivariate Bayesian modeling approach further enabled simultaneous examination of multiple engagement dimensions across professional subgroups, providing a more integrated understanding of thematic engagement heterogeneity in short-form vaccine communication.

Prior research suggests that appropriate, professionally grounded health communication may improve vaccine-related knowledge and risk perception, thereby supporting vaccination intention and uptake [20,63,64]. Building on this established evidence, our findings point to clear actionable directions for optimizing short-form video vaccine communication in China. Overall, improving vaccine education on short-form video platforms may require strategic thematic prioritization rather than simply increasing information volume. Medical professional producers may consider rebalancing content away from repeatedly covering system-oriented topics and toward clear responses to under-supplied but high-demand information on safety risks and side effects and management. This audience-centered approach leverages engagement feedback rather than assuming that informational completeness alone ensures public resonance [20]. Discrepancies between content supply and engagement outcomes highlight the value of data-driven feedback mechanisms. Continuous monitoring of theme-specific engagement patterns can generate actionable insights into audience preferences. Such monitoring may enable professional communicators to dynamically adjust content strategies and improve the effectiveness of vaccine education efforts in algorithm-driven short-form video environments [20]. Such prioritization does not imply abandoning scientific accuracy; rather, it suggests presenting professionally grounded information in an order and framing that better corresponds to audience concerns.

Several limitations should be acknowledged. First, engagement metrics primarily capture observable audience interaction and engagement-based responsiveness rather than actual vaccination attitudes or behavioral outcomes. In addition, these metrics may also be influenced by platform recommendation mechanisms and broader attention dynamics within algorithm-driven media environments. Second, no systematic adjustment was performed for variation in production quality, content style, or platform-specific recommendation algorithms and user demographics, all of which may shape content visibility and engagement outcomes [28,49]. Third, although the coding process underwent a preliminary consistency test, formal intercoder reliability statistics were not reported, and the findings should therefore be interpreted with consideration of potential coding variability.

Future research could adopt intervention-oriented designs, such as A/B testing of topic framing strategies, to evaluate how alternative presentations influence engagement with vaccine information on short-form video platforms. Particular attention should be given to identifying the causal effects of risk-based framing versus system-oriented information. Moreover, platform-specific strategy design is needed to account for variations in recommendation algorithms and user demographics. Such work would generate stronger causal evidence and more actionable guidance for optimizing professionally produced vaccine content.

## 5. Conclusions

This study examined the alignment between vaccine communication themes and engagement-based audience demand on short-form video platforms and identified structural mismatches in content produced by professional sources. These mismatches appear to stem not from a lack of information, but from a mismatch between professional thematic priorities and audience concerns in short-form video environments. Building on this insight, aligning thematic priorities with audience concerns may enhance the reach and practical value of professionally produced vaccine content.

## Figures and Tables

**Table 1:** Distribution of user engagement metrics by account type.

Account Type	Likes Median (IQR)	Shares Median (IQR)	Favorites Median (IQR)	Comments Median (IQR)
All videos	61 (17–460)	13 (1–190)	9 (1–119.2)	7 (2–51)
Medical professional	190 (68–2498)	63 (9–1582)	51 (8–1002)	21 (5–229.5)
Medical institution official media	25 (14–67)	5 (1–43)	4 (1–18)	2 (1–7)
Individual user	45 (13–301.5)	7 (0–130.5)	6 (1–81)	6 (2–38.5)
Private sector	70 (15–715)	27 (2–377)	17 (2–160.5)	5 (1–52)
Non-medical institution official media	45.5 (16–237.8)	12 (2–78.75)	6 (1–38.75)	3 (1–17)

Note: Values are presented as median (interquartile range).

**Table 2:** Supply–demand mismatch indices of content themes across account types.

Account Type	Weighted Demand Coverage (%)	Weighted Avoidance Coverage (%)	Demand Gap Index	Avoidance Redundancy Index	Overall Mismatch Index
Medical professional	15.5	34.8	0.026	0.722	0.304
Medical institution official media	16.2	40.8	0.000	0.942	0.377
Individual user	11.4	16.6	0.171	0.043	0.120
Private sector	13.2	17.5	0.108	0.073	0.094
Non-medical institution official media	13.0	15.5	0.114	0.000	0.068

Note: Weighted demand coverage and weighted avoidance coverage represent the weighted proportions of content themes classified as audience demand or avoidance within each account type, with greater emphasis placed on demand-related signals in index construction. Demand gap and avoidance redundancy indices summarize the relative imbalance in theme coverage, and the overall mismatch index combines both dimensions, with higher values indicating greater mismatch between content supply and audience engagement. Detailed calculation procedures are provided in Supplementary Method S2.

## Data Availability

The data used in this study were collected from publicly accessible social media platforms. Due to the substantial effort involved in constructing the curated dataset, the full dataset cannot be publicly shared. Aggregated data and analytic code of this study are available from the corresponding author upon reasonable request.

## Supplementary Materials

The following supporting information can be downloaded at: https://www.mdpi.com/article/10.3390/vaccines14060491/s1, Method S1: Bayesian negative binomial regression model specification, Method S2: Construction of Demand/Avoidance Levels and Mismatch Index, Table S1: Search terms used for video retrieval, Table S2: Coding scheme for video content themes, Table S3: Multivariate Bayesian negative binomial regression for engagement metrics among full dataset, Table S4: Multivariate Bayesian negative binomial regression for engagement metrics among videos posted by medical professionals, Table S5: Multivariate Bayesian negative binomial regression for engagement metrics among videos posted by medical institution official media, Figure S1: Posterior estimates of predictor effects on engagement across different account types.

## Abbreviations

The following abbreviations are used in this manuscript:

HBM	Health Belief Model
WHO	World Health Organization
BeSD	Behavioral and Social Drivers
CDC	Centers for Disease Control and Prevention
NIP	National Immunization Program vaccines
