# Supplementary material for: The Mismatch Between Professionally Produced Vaccine Content and Audience Demand on Chinese Short-Form Video Platforms: A Cross-Platform Content Analysis
Source: Vaccines (Basel). 2026 May 30;14(6):491. doi: 10.3390/vaccines14060491 (PMC13307767; doi:10.3390/vaccines14060491)
Supplement: Supplementary file 1 [file vaccines-14-00491-s001.zip › vaccines-4284766-supplementary.pdf]

Supplementary Materials

Method S1. Bayesian negative binomial regression model specification 2
Method S2. Construction of Demand/Avoidance Levels and Mismatch Index 2
Table S1. Search terms used for video retrieval 4
Table S2. Coding scheme for video content themes 4
Table S3. Multivariate Bayesian negative binomial regression for engagement metrics among full dataset 6
Table S4. Multivariate Bayesian negative binomial regression for engagement metrics among videos posted by medical professionals 6
Table S5. Multivariate Bayesian negative binomial regression for engagement metrics among videos posted by medical institution official media 7
Figure S1. Posterior estimates of predictor effects on engagement across different account types 8

Method S1. *Bayesian negative binomial regression model specification*

To examine the association between video content themes and user engagement, we fitted a Bayesian negative binomial regression model. Engagement metrics, including likes, shares, favorites, and comments, were treated as count outcomes reflecting user interaction with each video. A multivariate modeling framework was used in which four engagement indicators, including likes, favorites, shares, and comments, were jointly modeled while controlling for account-level covariates.

All models were estimated using four Markov chains with 4000 iterations each (2000 warm-up), resulting in 8000 posterior draws. Convergence diagnostics indicated satisfactory model convergence (R-hat ≈ 1.00 for all parameters).

The estimated overdispersion parameters were 0.25 (likes), 0.14 (shares), 0.16 (favorites), and 0.19 (comments). Regression coefficients (β) represent effects on the log-incidence rate ratio scale because a log link function was used. Positive coefficients indicate increased engagement rates relative to the reference category, whereas negative coefficients indicate decreased engagement.

Method S2. *Construction of Demand/Avoidance Levels and Mismatch Index*

Definitions of Demand or Avoidance levels

To evaluate the alignment between professional content supply and audience engagement demand, this study constructed a demand-avoidance match method based on engagement signals derived from Bayesian negative binomial regression models. User engagement responses (likes, shares, favorites, and comments) were interpreted as behavioral indicators reflecting audience interest or avoidance toward specific content themes. Positive regression coefficients (β) were interpreted as signals of audience demand, whereas negative coefficients were interpreted as audience avoidance signals.

Since engagement behaviors vary in magnitude and reflect different engagement patterns, demand and avoidance levels were not determined by a single indicator. Instead, two indicators were used to characterize the consistency and strength of engagement responses across four engagement outcomes in a multivariate Bayesian negative binomial regression model: (1) the number of positive or negative associations between the theme and engagement indicators ; (2) the average effect size, defined as the mean absolute value of the regression coefficients.

Based on these two indicators, themes were classified into three levels of demand or avoidance. The positive coefficients were interpreted as demand signals, while negative coefficients were interpreted as avoidance signals. Classification was based on the following criteria:

- High demand/avoidance: number of positive/negative associations ≥ 2 and average absolute effect size ≥ 0.2.
- Moderate demand/avoidance: number of positive/negative associations ≥ 2 or average absolute effect size ≥ 0.2.
- Low demand/avoidance: number of positive/negative associations < 2 and average absolute effect size < 0.2.

The threshold of 0.2 for the average effect size was selected as a pragmatic and data-informed criterion to differentiate levels of engagement relevance, based on the distribution of model estimates and to enable meaningful categorization into three levels.

Weighted coverage of demand and avoidance labels

To further quantify the alignment between professional information supply and audience engagement-based demand, a series of indicators was constructed, reflecting the extent to which supplied themes correspond to audience engagement preferences.

To quantify the supply of content themes, weighted demand/avoidance coverage was calculated according to the degree of demand or avoidance. The presence of the content themes was coded as a binary indicator (1 = present, 0 = absent) for each video. The mean value of this binary variable is regarded as the theme coverage rate. Demand themes were assigned weights of 1.0, 0.7 and 0.4 respectively from the highest to the lowest, while avoidance themes were assigned weights of 1.0, 0.6 and 0.3 respectively from the highest to the lowest. Weights were assigned to reflect the relative emphasis of the study, with greater importance placed on demand-related signals compared to avoidance. The weighted coverage score for each theme was calculated as the proportion of videos assigned to that theme multiplied by the corresponding weight.

Construction of supply-demand indices

To assess the degree of supply-demand gap in relation to audience demand, two types of gap measures were calculated within each thematic group.

The relative weighted gap was defined as:

$$\text{Relative Gap} = \frac{|\text{Max Coverage} - \text{Theme Coverage}|}{\text{Max Coverage}} \tag{1}$$

where Max Coverage represents the highest weighted demand or avoidance coverage within the group and Theme Coverage represents the weighted coverage of the focal theme. And an absolute weighted gap was calculated as:

$$\text{Absolute Gap} = \frac{|\text{Max Coverage} - \text{Theme Coverage}|}{100} \tag{2}$$

The final Demand Gap Index and Avoidance Redundancy Index were obtained by averaging the relative and absolute gap measures:

$$\text{Demand Gap Index} = \frac{\text{Relative Demand Gap} + \text{Absolute Demand Gap}}{2} \tag{3}$$

$$\text{Avoidance Redundancy Index} = \frac{\text{Relative Avoidance Gap} + \text{Absolute Avoidance Gap}}{2} \tag{4}$$

Finally, the Overall Mismatch Index was calculated as a weighted combination of the demand and avoidance indices:

$$\text{Overall Mismatch Index} = 0.6 \times \text{Demand Gap Index} + 0.4 \times \text{Avoidance Redundancy Index} \tag{5}$$

Demand was given a slightly higher weight because the study focused primarily on identifying unmet audience information needs rather than content redundancy.

Higher mismatch values indicate a greater discrepancy between the thematic distribution of supplied content and audience engagement-based demand.

Table S1. Search terms used for video retrieval

Category	Search Terms
General vaccine terms	疫苗 (Vaccine)，疫苗接种 (Vaccination)
Vaccine characteristics	疫苗副作用 (Vaccine side effects)，疫苗安全性 (Vaccine safety)，疫苗有效性 (Vaccine effectiveness)
Vaccine development and technologies	疫苗研发过程 (Vaccine development process)，灭活疫苗 (Inactivated vaccine)，腺病毒载体疫苗 (Adenovirus vector vaccine)，重组蛋白疫苗 (Recombinant protein vaccine)，减毒活疫苗 (Live attenuated vaccine)
Specific vaccines	HPV疫苗 (HPV vaccine)，九价疫苗 (9-valent HPV vaccine)，流感疫苗 (Influenza vaccine)，新冠疫苗 (COVID-19 vaccine)，儿童疫苗 (Childhood vaccine)，肺炎疫苗 (Pneumococcal vaccine)

Table S2. Coding scheme for video content themes

**Theory**	**Classification**	**Theme**	**Description**
Health Belief Model	Risk Perception	Susceptibility	Discusses the epidemiology of disease, their causes, at-risk groups, and prevention
		Severity	Focuses on symptoms, diagnosis, treatment, and prognosis of diseases
	Perceived Utility & Scientific Understanding	Effectiveness	Talks about the positive impact of vaccines on preventing diseases
		Mechanism of action	Describes how vaccines work to provide immunity
	Safety & Risk management	Safety	Assures the safety of vaccines and their lack of harmful side effects
		Side Effects & Management	Provides information on potential side effects and how to manage them
Behavioral and Social Drivers	Structural & Practical Factors	Accessibility & Affordability	Information on how and where to access vaccines and their cost
		Procedure	Details about the vaccine schedule, required doses, and suitable population
		Precautions	Discusses considerations and recommended practices across the entire vaccination process

Health Misinformation Theory and Social Amplification of Risk Framework	Information Environment	Negative Reviews & Health Myths	Criticism or complaints about vaccines, including the spread of misinformation

Table S3. *Multivariate Bayesian negative binomial regression for engagement metrics among full dataset*

Variable		Likes β (95% CrI)	Shares β (95% CrI)	Favorites β (95% CrI)	Comments β (95% CrI)
Content theme	Susceptibility	0.25 (0.12, 0.39)	—	0.19 (0.03, 0.37)	0.22 (0.07, 0.37)
	Effectiveness	—	-0.14 (-0.28, -0.01)	—	-0.28 (-0.39, -0.16)
	Mechanism of action	—	—	0.49 (0.09, 0.92)	—
	Safety	—	—	-0.56 (-0.95, -0.14)	—
	Accessibility & Affordability	—	—	0.21 (0.06, 0.36)	—
	Precautions	-0.27 (-0.42, -0.13)	—	—	-0.27 (-0.43, -0.12)
	Side effects & Management	0.31 (0.10, 0.53)	—	0.39 (0.11, 0.68)	0.67 (0.44, 0.91)
	Negative reviews & health myths	—	0.42 (0.13, 0.73)	—	—
Video type	Health education	-0.32 (-0.58, -0.06)	-0.31 (-0.67, 0.03)	-0.53 (-0.88, -0.19)	-0.03 (-0.33, 0.25)
	Hot topic news	-0.33 (-0.62, -0.05)	-0.12 (-0.51, 0.25)	-1.04 (-1.40, -0.69)	0.41 (0.09, 0.72)
	Lifestyle sharing	-0.73 (-1.02, -0.45)	-1.07 (-1.44, -0.71)	-1.19 (-1.55, -0.84)	-0.26 (-0.58, 0.05)
Vaccine category	NIP vaccines	0.65 (0.42, 0.90)	0.66 (0.35, 0.97)	0.38 (0.08, 0.68)	0.87 (0.61, 1.12)
	Non-NIP vaccines	0.07 (-0.10, 0.24)	0.32 (0.09, 0.54)	-0.17 (-0.40, 0.05)	0.39 (0.20, 0.58)
	Both NIP and Non-NIP vaccines	0.69 (0.39, 0.99)	1.04 (0.66, 1.45)	1.28 (0.90, 1.67)	0.36 (0.03, 0.70)
Account type	Non-medical institutional media	-1.02 (-1.24, -0.80)	-0.71 (-1.01, -0.40)	-0.76 (-1.02, -0.49)	-0.88 (-1.14, -0.62)
	Private sector	0.29 (-0.20, 0.84)	0.13 (-0.51, 0.86)	0.30 (-0.30, 0.98)	-0.55 (-1.10, 0.06)
	Medical professionals	0.39 (0.19, 0.59)	0.85 (0.59, 1.12)	0.93 (0.68, 1.19)	0.31 (0.08, 0.54)
	Medical institution official media	-1.60 (-1.94, -1.22)	-0.06 (-0.53, 0.47)	-1.30 (-1.75, -0.83)	-1.44 (-1.84, -1.01)
Social influence		0.76 (0.69, 0.83)	0.55 (0.45, 0.65)	0.67 (0.57, 0.76)	0.74 (0.66, 0.82)

Table S4. *Multivariate Bayesian negative binomial regression for engagement metrics among videos posted by medical professionals*

Variable		Likes β (95% CrI)	Shares β (95% CrI)	Favorites β (95% CrI)	Comments β (95% CrI)
Content theme	Susceptibility	0.40 (0.21, 0.60)	0.49 (0.23, 0.76)	0.63 (0.39, 0.89)	—
	Effectiveness	—	—	—	0.18 (0.01, 0.36)
	Mechanism of action	-1.26 (-1.70, -0.78)	-2.06 (-2.61, -1.48)	-1.69 (-2.20, -1.13)	-1.66 (-2.12, -1.17)
	Safety	—	0.87 (0.15, 1.69)	—	0.57 (0.04, 1.15)

	Accessibility & Affordability	0.64 (0.35, 0.95)	0.74 (0.40, 1.12)	0.95 (0.62, 1.32)	0.37 (0.08, 0.70)
	Precautions	-0.25 (-0.48, -0.02)	—	-0.37 (-0.63, -0.11)	—
	Negative reviews & health myths	—	—	—	0.94 (0.34, 1.61)
Video type	Health education	-2.18 (-2.84, -1.59)	-2.24 (-3.27, -1.26)	-1.43 (-2.18, -0.76)	-1.64 (-2.39, -0.97)
	Hot topic news	-1.44 (-2.25, -0.65)	-0.82 (-2.00, 0.36)	-1.14 (-2.09, -0.20)	-0.72 (-1.71, 0.22)
	Lifestyle sharing	-2.92 (-3.90, -1.88)	-2.49 (-3.90, -1.01)	-2.24 (-3.41, -0.96)	-2.05 (-3.19, -0.87)
Vaccine category	NIP vaccines	-1.02 (-1.44, -0.59)	-1.50 (-2.04, -0.94)	-1.24 (-1.75, -0.72)	-0.59 (-1.06, -0.11)
	Non-NIP vaccines	-1.18 (-1.51, -0.86)	-1.63 (-2.06, -1.22)	-1.84 (-2.25, -1.44)	-1.14 (-1.50, -0.79)
	Both NIP and Non-NIP vaccines	-0.62 (-1.25, 0.05)	-1.01 (-1.80, -0.13)	-0.75 (-1.48, 0.05)	-0.83 (-1.53, -0.08)
Social influence		1.49 (1.33, 1.65)	1.62 (1.37, 1.85)	1.58 (1.38, 1.78)	1.43 (1.26, 1.61)

Table S5. *Multivariate Bayesian negative binomial regression for engagement metrics among videos posted by medical institution official media*

	Variable	Likes β (95% CrI)	Shares β (95% CrI)	Favorites β (95% CrI)	Comments β (95% CrI)
Content theme	Severity	—	1.03 (0.49, 1.58)	—	—
	Susceptibility	0.68 (0.23, 1.16)	—	—	—
	Effectiveness	—	—	—	-0.54 (-0.92, -0.14)
	Side effects & Management	1.19 (0.15, 2.32)	—	—	2.11 (1.38, 2.92)
Video type	Health education	0.87 (-0.48, 1.95)	2.09 (-0.60, 4.07)	1.87 (-0.50, 3.66)	-0.35 (-2.01, 1.01)
	Hot topic news	0.28 (-1.21, 1.57)	1.86 (-1.04, 4.28)	0.85 (-1.86, 3.25)	-0.93 (-2.83, 0.70)
	Lifestyle sharing	5.85 (3.78, 8.44)	10.49 (6.64, 15.66)	6.60 (3.05, 11.51)	4.86 (2.29, 8.17)
Vaccine category	NIP vaccines	-1.07 (-2.31, 0.16)	-1.87 (-3.34, -0.29)	-2.78 (-4.10, -1.41)	—
	Non-NIP vaccines	-1.84 (-2.95, -0.76)	-4.39 (-5.62, -3.25)	-3.53 (-4.64, -2.50)	—
	Both NIP and Non-NIP vaccines	-1.34 (-2.71, 0.08)	-3.82 (-5.52, -1.94)	-2.97 (-4.48, -1.25)	—
Social influence		0.87 (0.46, 1.31)	0.87 (0.00, 1.85)	0.62 (0.04, 1.24)	1.30 (0.77, 1.86)

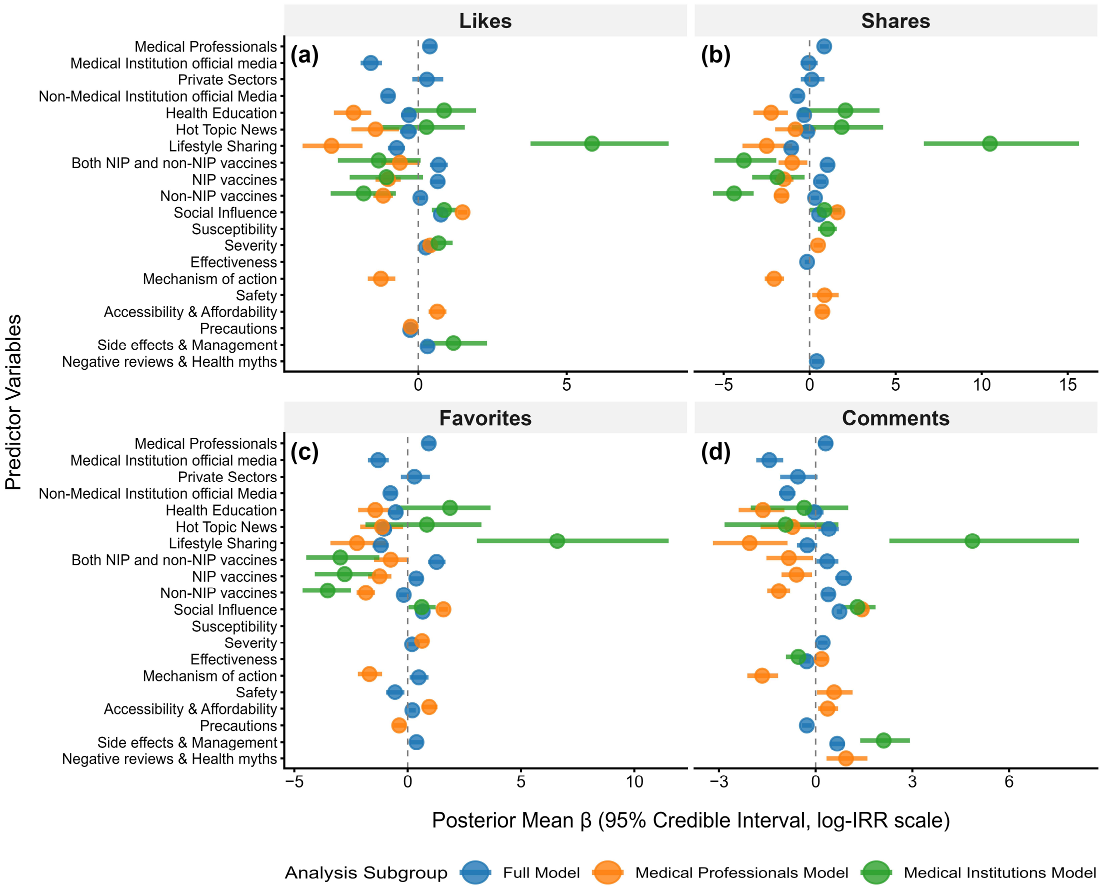

Figure S1. *Posterior estimates of predictor effects on engagement across different account types*

Forest plots show posterior mean coefficients and 95% credible intervals from multivariate Bayesian negative binomial regression models predicting four engagement metrics: (a) likes, (b) shares, (c) favorites, and (d) comments. The dashed vertical line represents the null effect ($\beta = 0$), where pcoefficients to the right indicate higher expected engagement compared with the reference category. Points are offset horizontally to avoid overlap between subgroups.
